# A combination of incidence data and mobility proxies from social media predicts the intra-urban spread of dengue in Yogyakarta, Indonesia

**DOI:** 10.1371/journal.pntd.0007298

**Published:** 2019-04-15

**Authors:** Aditya Lia Ramadona, Yesim Tozan, Lutfan Lazuardi, Joacim Rocklöv

**Affiliations:** 1 Department of Public Health and Clinical Medicine, Section of Sustainable Health, Umeå University, Umeå, Sweden; 2 Center for Environmental Studies, Universitas Gadjah Mada, Yogyakarta, Indonesia; 3 College of Global Public Health, New York University, New York, United States of America; 4 Department of Health Policy and Management, Faculty of Medicine, Universitas Gadjah Mada, Yogyakarta, Indonesia; Universidade do Estado do Rio de Janeiro, BRAZIL

## Abstract

Only a few studies have investigated the potential of using geotagged social media data for predicting the patterns of spatio-temporal spread of vector-borne diseases. We herein demonstrated the role of human mobility in the intra-urban spread of dengue by weighting local incidence data with geo-tagged Twitter data as a proxy for human mobility across 45 neighborhoods in Yogyakarta city, Indonesia. To estimate the dengue virus importation pressure in each study neighborhood monthly, we developed an algorithm to estimate a dynamic mobility-weighted incidence index (MI), which quantifies the level of exposure to virus importation in any given neighborhood. Using a Bayesian spatio-temporal regression model, we estimated the coefficients and predictiveness of the MI index for lags up to 6 months. Specifically, we used a Poisson regression model with an unstructured spatial covariance matrix. We compared the predictability of the MI index to that of the dengue incidence rate over the preceding months in the same neighborhood (autocorrelation) and that of the mobility information alone. We based our estimates on a volume of 1·302·405 geotagged tweets (from 118·114 unique users) and monthly dengue incidence data for the 45 study neighborhoods in Yogyakarta city over the period from August 2016 to June 2018. The MI index, as a standalone variable, had the highest explanatory power for predicting dengue transmission risk in the study neighborhoods, with the greatest predictive ability at a 3-months lead time. The MI index was a better predictor of the dengue risk in a neighborhood than the recent transmission patterns in the same neighborhood, or just the mobility patterns between neighborhoods. Our results suggest that human mobility is an important driver of the spread of dengue within cities when combined with information on local circulation of the dengue virus. The geotagged Twitter data can provide important information on human mobility patterns to improve our understanding of the direction and the risk of spread of diseases, such as dengue. The proposed MI index together with traditional data sources can provide useful information for the development of more accurate and efficient early warning and response systems.

## Author summary

Recent studies have shown that Twitter can be utilized as a tool for health research, and aggregated large-scale social media data can indicate the risk of infectious disease in real-time with high accuracy and at low cost. However, most of these studies relied primarily on content analysis or text mining, while only a few analyzed the networks of Twitter users. None has incorporated user geolocation data to explain health outcomes at an intra-urban level. Currently dengue early warning systems rely on syndromic surveillance, which lacks completeness and timeliness. Effective syndromic surveillance is rarely achieved due to its technical complexity and a general lack of capacity. Researchers have assessed vector indices, meteorological factors and environmental variables as predictors of dengue incidence, but have failed to capture the complexity of transmission as it relates to human behaviors and movements. Here we develop an algorithm to estimate a dynamic mobility-weighted incidence index (MI), which quantifies the level of exposure to virus importation in a given neighborhood. The proposed index is based on publicly available social media and routine disease surveillance data, and provides a low-cost source of information for assessing the risk of spread of communicable diseases, such as dengue. This study suggests that the MI index is of utility and significance for dengue surveillance and early warnings systems and can enhance timely decision-making within the public health system.

## Introduction

Dengue has become a major concern for public health authorities in tropical and sub-tropical developing countries [1]; the frequency and magnitude of epidemics, the incidence of severe disease, and the rate of hospitalizations have increased in the past few decades [2]. Asia-Pacific countries bear the heaviest disease burden of dengue where over 1·8 billion people are estimated to be at risk of infection [3,4]. Dengue also poses a serious economic challenge to countries due to high costs of dengue prevention and control programs, particularly during epidemic peaks [5,6].

Timely and accurate disease reporting and forecasting is the pillar of infectious disease control. However, public health agencies often report disease trends and outbreaks with severe delays, and reporting tends to be based on aggregated disease data at national or regional levels with little information about disease counts and trends at local levels. Dengue is a notifiable disease in most endemic countries; however, several studies revealed high levels of under-reporting in routine surveillance systems, particularly from ambulatory care settings [2]. These shortcomings hamper programmatic efforts on the ground to mount timely, context-specific, and effective response to abnormal disease events, including incipient epidemics [7].

Population growth, unplanned urbanization, increased vector density, and climate variability are all identified as important contributing factors to dengue propagation [8]. Spatial and temporal variation in interactions among hosts, dengue viruses, vectors and the environment have led to a heterogeneous distribution of dengue risk across geographical locations [9–11]. Understanding how these complex interactions influence the epidemiology of dengue at different spatial and temporal scales is important to assess transmission risk and allocate resources efficiently [8,12]. A main obstacle to studying such complex interactions has been the limited availability of large-scale spatial and temporal datasets.

Several studies have explored using near real-time streaming data from Twitter to investigate public health trends. As of the first quarter of 2017, there were about 328 million monthly Twitter users worldwide [13]. This large volume of social media data may be exploited for public health monitoring and surveillance purposes [14,15]. The most recent literature has focused on analysing Twitter content using text mining methods to estimate and forecast infectious disease activity [16,17], predict heart disease mortality [18], and measure health-related quality of life [19]. One study explored the use of Twitter content for dengue forecasting, but focused on verifying the correlation between number of dengue cases and dengue-related tweets posted over the same time period [20].

In this study, we investigated the use of publicly available geotagged Twitter data for predicting the spatio-temporal clustering patterns of dengue incidence. First, we designed, implemented and evaluated an algorithm that harvested and analysed real-time Twitter streams to estimate proxies of human mobility in a densely populated urban area. Then we weighted the incidence of dengue in all neighborhoods by the mobility proxies to specific locations and generated a dynamic Mobility-weighted Incidence (MI) index. Lastly, we demonstrated that the MI index was highly predictive of the temporal and spatial patterns of dengue spread in Yogyakarta municipality, Indonesia.

## Methods

### Data

**Fig 1:**
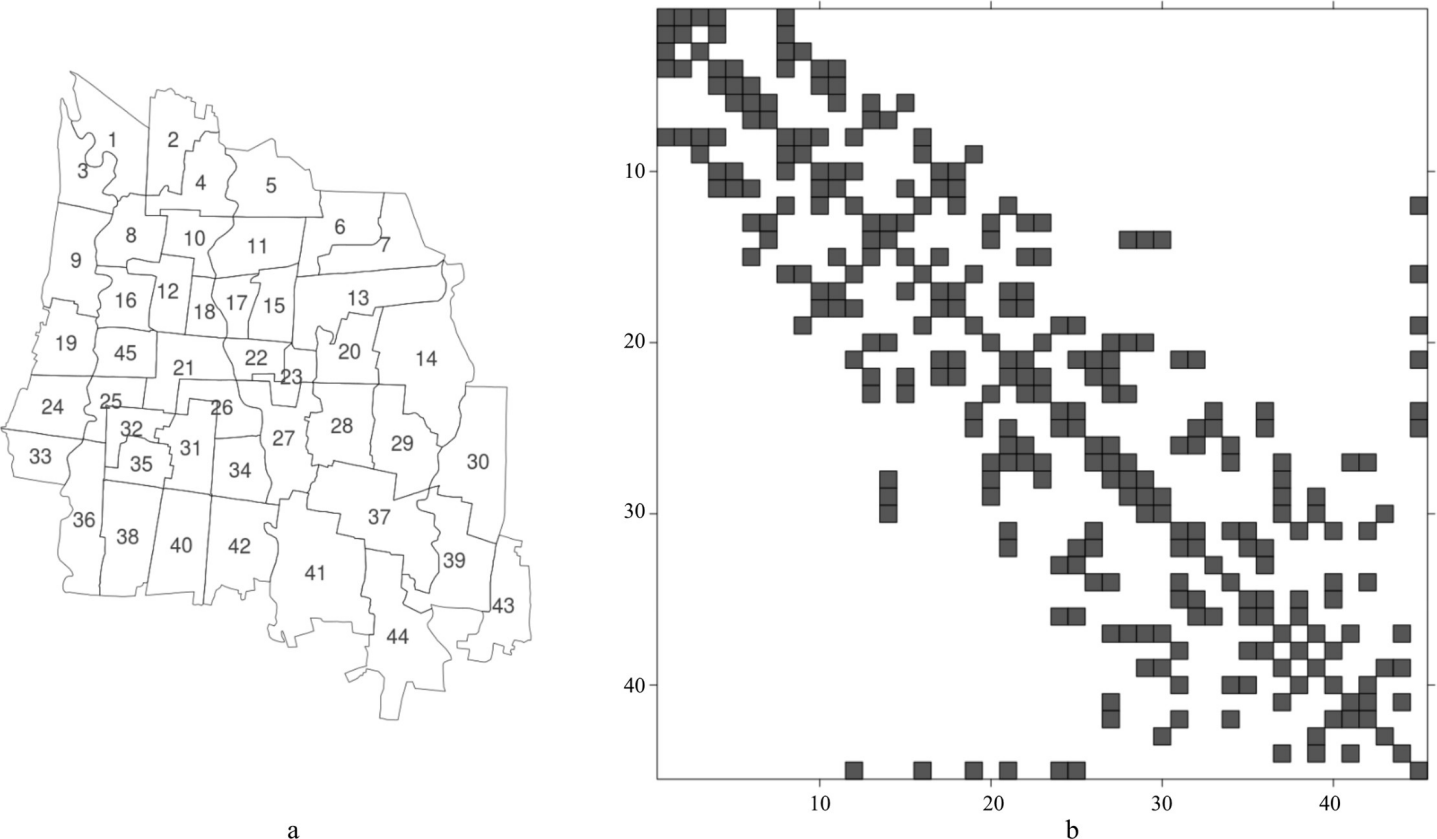
The map (panel a) and the adjacency matrix (panel b) of the 45 study neighborhoods (rows and columns identify areas; squares identify neighborhoods) in Yogyakarta municipality, Indonesia.

The study was conducted in Yogyakarta municipality, one of the five districts and the capital of Yogyakarta Province in Indonesia. Yogyakarta municipality is a medium sized, densely populated, and rapidly developing urban area, spread over 32.5 km^2^ with an average population density of 14,000 persons/km^2^. It is located about 538 km away from the capital Jakarta and lies between 75 to 132 m above sea level in the central southern part of Java island at 07°45’57”–07°50’25” S and 110°20’41”–110°24’14” E [21]. Yogyakarta municipality is divided into 45 neighborhoods (Fig 1, number 1 to 45), ranging in surface area between 0.3 and 1.68 km^2^. This study used neighborhoods as the geographical unit of observation.

We obtained monthly dengue cases (i.e. dengue fever, dengue haemorrhagic fever, and dengue shock syndrome) for each neighborhood (Den) during the period August 2016–June 2018 from the Dengue Surveillance Report of the Yogyakarta Municipality Health Office. We complemented dengue surveillance data with geotagged tweets posted in the administrative boundaries of the study area during the same period. To achieve this, we employed the Twitter’s Application Programming Interface (API) and selected Tweets within Yogyakarta municipality for analysis. We only extracted the user identification string, timestamp, and longitude and latitude of the user’s location in the Tweets. We overlaid the geotagged tweets on the administrative map of the study area and exchanged the geocode to the neighborhood identification number (ID).

We formulated an algorithm to estimate a dynamic MI index, quantifying the level of exposure to virus in any given study neighborhood due to importation form other neighborhoods. The MI index was calculated based on Twitter users’ mobility patterns between pairs of neighborhoods. The mobility patterns were computed by estimating the rate with which a Twitter user in one study neighborhood re-tweeted in another neighborhood within the same month. Based on this information, we generated a monthly matrix (I_t_) measuring the cumulative number of mobility events between each pair of neighborhoods at time *t*, in months. Then, we created the monthly mobility network (N_i,j,t_) of neighborhoods by multiplying (I_t_) with its transpose (I_t_^T^). We set the diagonal of the 45×45 matrix of the affiliation to zero. Then we standardized the monthly mobility matrix (N_i,j,t_) by dividing it by the total number of mobility events observed at time *t* (N_t_) for all the neighborhoods. This ensured that, at a fixed time, the mobility matrix would always sum to 1. We referred to the standardized mobility matrix as, Ň_t_. To capture the total exposure to incoming mobility into each neighborhood, *j*, we aggregated the standardized mobility from all the 45 neighborhoods over one month and referred to this as the TW_jt_. The TW index is thus a time dependent vector of length 45. We further constructed a new matrix by multiplying the standardized mobility, Ň_ijt_, by the vector of the number of dengue cases reported in each neighborhood *i* (of outgoing mobility), and we referred to this index as importations and computed it as I_ijt_ = Ň_ijt_ × Den_it_. Lastly, to capture the total exposure to the dengue virus imported due to human mobility into each neighborhood, *j*, from all the other neighborhoods, i, we aggregated the importations, I_ijt_, from all the 45 neighborhoods over one month and referred to this as the MI_jt_. The MI index is thus a time dependent vector of length 45.

We then investigated the association between dengue incidence and the Den, TW and MI variables using a Bayesian spatio-temporal modelling framework assuming a Poisson distribution of the monthly counts in each neighborhood. In the model, we estimated and adjusted for the spatial covariance between neighborhoods using an unstructured spatial covariance matrix. We further adjusted for the influence of population size variability across neighborhoods by offsetting population size. Thus, the regression analysis assessed predictors of the incidence of dengue. We implemented the models using the INLA R-package [22,23]. In the regression model, we started out by investigating how much of the variability in the dengue counts could be explained by the spatial covariance and intercept model only (the null model), leaving out all predictor variables. Subsequently, we included the MI, TW and Den variables one lag at a time (crude), and then all lags 1 to 6 months simultaneously, but only one variable at a time. For variables showing important prediction skill, we also analyzed their combined predictive ability. The models were fitted with all lags in the same model, but with only one of the MI, TW and Den variables at a time. The model structure can be described as:

*y_it_ = Poisson(λ_it_)*; *λ_it_ = E_it_ ρ_it_*

log(ρ_it_) = η_it_

η_it_ = b_o_+ Σ β_k_ z_i(t-k)_ + u_i_+v_i_ + log(p_i_)

The terms *u*_*i*_ and *v*_*i*_ are the spatial effects, representing unspecified features of neighborhood *i* that do and do not display spatial structure [24], respectively. The *k* indicates the lag in months and takes values from 1 to 6. The *z* corresponds to the variables MI, TW and Den. The coefficient *β*_*k*_ represents the regression coefficients for the variable *z* at lag *k*. The *p*_*i*_ variable offsets the population size of neighborhood *i*. The models were evaluated based on the Bayesian Information Criterion (BIC) and the estimate of R-square, as well as on prediction performance according to the standardized root mean square error (SRMSE).

## Results

**Fig 2:**
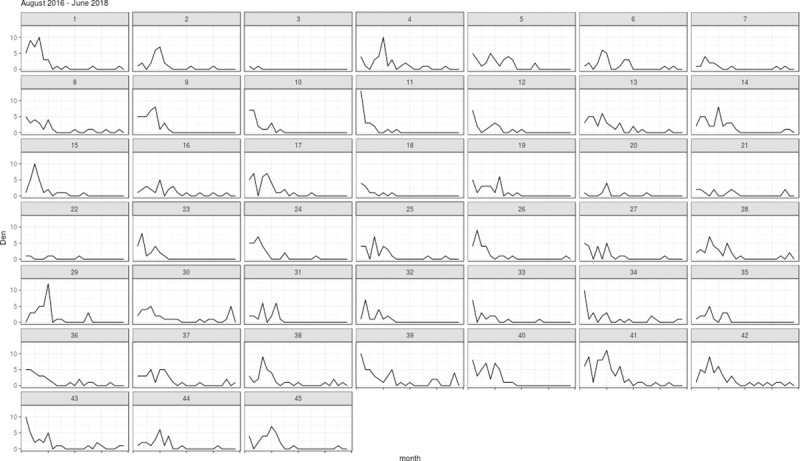
Time-series of reported dengue cases (Den) between August 2016 and June 2018 for the 45 study neighborhoods in Yogyakarta municipality, Indonesia.

The total number of dengue cases during the 23-month study period was 1,203, with the highest monthly count of 13 cases reported for neighborhood ID 11 in August 2016. The monthly incidence of dengue in the study area increased gradually from December to March of next year and then decreased until the start of the rainy season in October (Fig 2). Overall, the incidence of dengue was decreasing over the study period.

The number of Twitter users and the population size of each study neighborhood are shown in **S1 Fig**.

**Fig 3:**
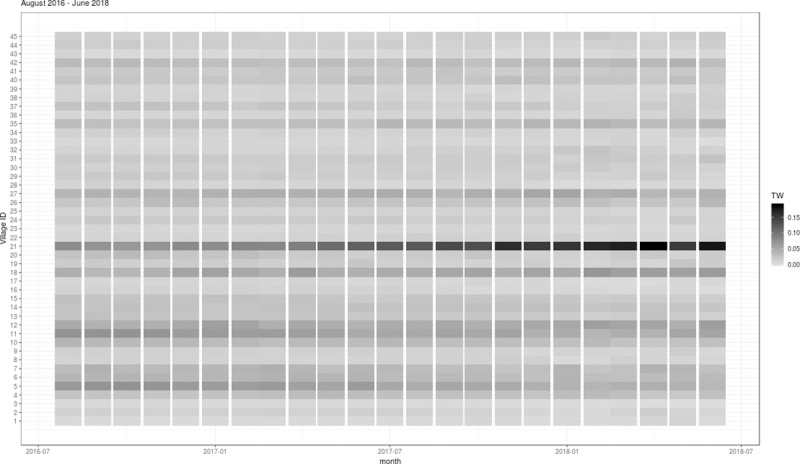
The TW index capturing the temporal pattern of the aggregated total monthly mobility into each of the 45 study neighborhoods in Yogyakarta municipality, Indonesia, August 2016—June 2018.

**Fig 4:**
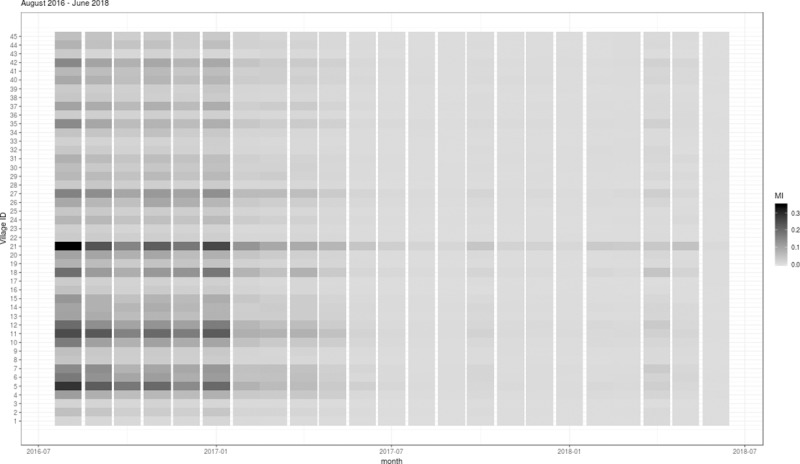
The MI index estimating the temporal pattern of the aggregated importations into each of the 45 study neighborhoods in Yogyakarta, Indonesia, August 2016—June 2018.

The monthly mobility patterns for the 45 neighborhoods appeared to be relatively consistent over the study period, except that a slight increase in the number of mobility events was observed over the same period. The mobility patterns varied considerably across different pairs of neighborhoods (Fig 3). The MI index (Fig 4) for each neighborhood reflected a combination of the mobility estimates and the disease counts (Fig 2). In general, we found that the MI index was not only higher for the neighborhoods with relatively higher mobility to other neighborhoods, but also reflected the decreasing trend in the disease counts over time (Figs 3 and 4).

**Fig 5:**
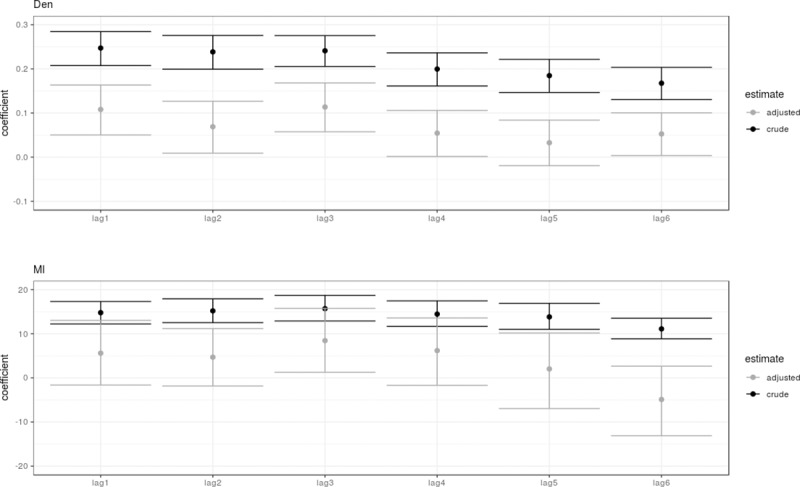
The crude and adjusted coefficients for the Den and MI models for lag times 1 to 6.

Table 1 describes the crude and adjusted model estimates of the lag effects of Den, TW and MI using the Bayesian spatio-temporal regression model. We found that the mobility and the centrality of a neighborhood proved not to be important for predicting the incidence of dengue on its own. This is shown by comparing the model fit of the TW lag variables (crude and adjusted) to the null model and their observed lack of difference in the R-square, BIC and SRMSE in Table 1. In contrast, the Den and MI variables provided important information for predicting the incidence of dengue at lead times 1 to 6 months based on the crude and adjusted estimates of the model (Table 1). The coefficients from the crude and adjusted models are graphically presented in Fig 5. Unsurprisingly, the uncertainty and confidence intervals for the coefficient estimates increased in the lag adjusted models compared to the crude single lag models. Overall, the coefficients were smaller in the adjusted models. This is because of the similarity of information carried over in lags of a specific variable, i.e. due to temporal covariance. In the adjusted models, we observed a decreasing pattern in the association to the Den and MI variables with increasing lags, with the exception that both peaked at lag 3 months. While most lags associated with the Den variable showed statistically significant associations, the associations with the MI variable were more uncertain, with the exception of at lag 3 months. However, since the SRMSE was lower for the MI model, it appeared that this variable still included more vital information for predicting the incidence of dengue in the neighborhoods. Furthermore, an inspection of the crude estimates strongly supported this conclusion, where the MI variable at lag 3 months had clearly the best predictive ability and almost the same predictive ability as the adjusted models with all lags, in view of the R-square, BIC and SRMSE values (Table 1). The Den variable at lag 3 months did not show a similar good performance with significantly lower predictive ability, R-square, BIC and SRMSE.

**Table 1:** Model fitting statistics and coefficients (R-sq = R square, BIC = Bayesian Information Criterion, SRMSE = standardized root mean square error, coefficient mean = log(relative risk), coefficient sd = standard error, 2.5 percentile = lower end of 95% credible interval of coefficient, 97.5 percentile = higher end of 95% credible interval of coefficient).

Variable	R-sq	BIC	SRMSE	fixed effects			
				coefficient mean	coefficient sd	2.5 percentile	97.5 percentile
Null model	0.064	1337.855	0.882	-10.0739	0.0668	-10.2084	-9.9463
**Crude lag estimates**							
Den lag1	0.098	1246.184	0.865	0.2472	0.0196	0.2075	0.2844
Den lag2	0.123	1244.557	0.854	0.2385	0.0194	0.1993	0.2757
Den lag3	0.164	1213.810	0.833	0.2410	0.0178	0.2054	0.2754
Den lag4	0.090	1262.592	0.869	0.1996	0.0191	0.1612	0.2363
Den lag5	0.105	1270.408	0.862	0.1847	0.0191	0.1465	0.2215
Den lag6	0.080	1278.772	0.874	0.1675	0.0186	0.1304	0.2034
TW lag1	0.065	1337.276	0.881	4.2158	2.9655	-1.8731	9.8080
TW lag2	0.065	1337.581	0.881	4.0453	3.0282	-2.1836	9.7437
TW lag3	0.065	1337.596	0.881	4.2636	3.1498	-2.1978	10.2092
TW lag4	0.066	1337.292	0.881	4.7574	3.2264	-1.8396	10.8690
TW lag5	0.066	1336.294	0.881	5.7825	3.2885	-0.9000	12.0587
TW lag6	0.066	1336.181	0.881	6.0791	3.3850	-0.7870	12.5534
MI lag1	0.086	1237.947	0.871	14.7771	1.2940	12.2140	17.3077
MI lag2	0.132	1224.506	0.849	15.1857	1.3731	12.5236	17.9179
MI lag3	0.197	1198.824	0.817	15.6868	1.4773	12.8975	18.7013
MI lag4	0.174	1221.191	0.828	14.4542	1.4724	11.6631	17.4517
MI lag5	0.172	1220.364	0.829	13.8227	1.4964	11.0159	16.8935
MI lag6	0.137	1230.597	0.847	11.1021	1.1871	8.8499	13.5165
**Adjusted lag estimates**							
Den lag1 Den lag2 Den lag3 Den lag4 Den lag5 Den lag6	0.201	1168.257	0.815	0.1080 0.0689 0.1136 0.0544 0.0326 0.0526	0.0288 0.0300 0.0282 0.0266 0.0263 0.0247	0.0503 0.0090 0.0574 0.0015 -0.0193 0.0035	0.1634 0.1267 0.1681 0.1058 0.0839 0.1005
TW lag1 TW lag2 TW lag3 TW lag4 TW lag5 TW lag6	0.068	1340.723	0.880	0.1794 -9.0344 -10.4439 -4.4227 15.4022 15.9111	12.6758 14.7070 15.8923 15.8500 15.9844 14.9473	-24.7466 -38.1347 -41.8313 -35.7277 -16.1796 -13.5140	25.0110 19.6128 20.5637 26.5013 46.5789 45.1656
MI lag1 MI lag2 MI lag3 MI lag4 MI lag5 MI lag6	0.217	1194.667	0.807	5.6002 4.6889 8.4452 6.1895 2.0293 -4.8919	3.7329 3.3112 3.6902 3.8815 4.3635 4.0121	-1.6199 -1.8357 1.2679 -1.6790 -6.9640 -13.1124	13.0294 11.1655 15.7461 13.5752 10.1850 2.6493
**Combined variable model**							
Den lag1 Den lag2 Den lag3 Den lag4 Den lag5 Den lag6 MI lag1 MI lag2 MI lag3 MI lag4 MI lag5 MI lag6	0.271	1140.838	0.778	0.0816 0.0485 0.0964 0.0510 0.0265 0.0445 3.2332 3.8463 7.0018 2.9156 0.5181 -4.5618	0.0302 0.0302 0.0279 0.0273 0.0269 0.0246 3.9101 3.6771 3.9220 4.1978 4.3102 3.9001	0.0213 -0.0118 0.0408 -0.0034 -0.0267 -0.0045 -4.3982 -3.4612 -0.7220 -5.6567 -8.3993 -12.5642	0.1398 0.1068 0.1505 0.1040 0.0788 0.0921 10.9449 10.9795 14.6739 10.8263 8.5290 2.7519

The model including both the Den and MI variables at lags 1 to 6 months estimated an R-square, BIC and SRMSE of 0.271, 1140.8 and 0.778, respectively, and showed considerably higher predictive ability compared to the adjusted models of the Den and MI variables alone (Table 1). This supports the fact that these variables contributed different information to the predictive ability of the model. Looking at the coefficients in this combined model, the estimates were not very different than those obtained from the adjusted single variable model estimates, confirming the exclusive unique contribution of these two variables to the predictive ability.

## Discussion

This study revealed insights into how the intra-urban outbreak risk relates to a combination of human mobility and the size of local outbreaks, and developed a new early warning variable indicating the risk of spread. The indicator integrated human mobility proxies derived through an analysis of Twitter user geolocation data with disease surveillance data, and demonstrated its ability as a predictor of dengue incidence up to 6 months lead time at the intra-city level. The proposed MI index captures dynamic network properties in a simplified and condensed form and can be used in regression models, similar to the model fitted here, to describe complex spatio-temporal interactions between human mobility and disease spread. We found that the impact of human mobility on disease spread cannot be effectively studied without combining mobility information with disease incidence data. This is not surprising because mobility does not necessarily translate into a greater exposure to the circulating virus unless it is combined with disease incidence information—this is exactly what the new MI index captures. We propose further the development of methods and the testing of the MI index, particularly for predicting the risk of incidence and spread of dengue with a lead time of 3 months. We also propose that future research should consider the combined effects of the MI index and the past cases in the same location (the Den variable), which was found to contribute significantly to the prediction accuracy of the models. These findings have implications for empirical studies assessing the incidence risk (such as adjusting for mobility bias in cluster randomized trials) and for risk assessments at both micro and macro geographical levels, especially in the development of early warnings systems using near-real time data [25,26,27].

The demonstrated predictive ability of the MI index alone (20% of the variability in the incidence of dengue in mutually exclusive locations) and in combination with auto-correlative terms (27% of the variability in the incidence of dengue in mutually exclusive locations) hold great promise for improving predictions, early warning systems, and timely response. It also highlights the importance of understanding better the role of population mobility in the spread of arboviruses at the intra-city scale. The combined use of autoregressive terms and the MI index along with other factors, such as weather variability, environmental characteristics, and vector activity, is likely to yield substantially improved predictions. Furthermore, adjusting for virus exposure using the MI index would be important for studies mapping the spatial and spatiotemporal risk factors for dengue. For instance, human mobility, as shown in this study, is an important predictor and a potential confounder of the local incidence of dengue at the spatiotemporal scales.

This analysis benefited from a novel data source and a novel procedure for tracking and predicting human mobility from publicly available social media data, providing a low-cost source of information. Given the high explanatory power of the MI index to describe the variability in dengue incidence, we believe that social media driven mobility indicators have the potential to allow researchers to assess the risk of communicable diseases, such as dengue, in real time by capturing dynamic network properties of importance for timely disease control.

We estimated the user mobility patterns and the affiliation network in a relatively small but densely populated urban area by utilizing data from the Twitter’s API. The retrieved data from the API represent only about 1% of the Twitter volume, but previous research suggests that when geographic boundary boxes are used almost the complete sample of Twitter location data can be extracted [28,29]. Ideally, it is better to use data from Twitter’s Firehose. The major drawbacks of Firehose data are its prohibitively high cost and large storage and computational resource requirements [29], both of which can adversely affect the sustainability of translational applications of such data for public health preparedness and response.

We derived mobility from a rather short (23-month) time-series data from August 2016 to June 2018 to infer for the degree of association between the MI index and the observed dengue cases. Future studies should assess the predictive performance of the MI index further by using longer prospective validation series and building more complete models of dengue disease dynamics by including other predictive factors. We further suggest that future studies investigate non-linearities in virus exposure and response relationships and implement a distributed lag approach. Despite these limitations, we were able to demonstrate a strong association of the MI index with reported dengue cases. Therefore, we believe that the MI index holds promise as an alarm variable in disease surveillance and early warning systems, contributing to a better understanding of spatial patterns of outbreak clusters over time, namely dynamic hotspots.

A limitation of this study is the assumption that user movements between consecutive tweets were representative of the overall population mobility, while in fact Twitter users may represent a selected group of individuals. It is, however, important to note that the use of Twitter and other social media platforms is very common in Indonesia [30], and that the demonstrated predictive ability of the MI index in this study supports the belief that Twitter data can capture the important aspects of mobility relevant for the spread of dengue in a densely populated urban area. This goes hand in hand with prior studies validating Twitter as a viable data source to study human mobility [31,32]. Using mobile phone data with geo-tags would have been a better alternative, although the downside is that such data are harder to acquire and use prospectively over time. Yet, human mobility patterns extracted from geotagged tweets have been reported to have similar overall features with mobile phone records [33].

The analysis employed a novel procedure for tracking and predicting human mobility and dengue spread at the intra-urban level using publicly available social media data from Twitter. We demonstrated that dengue cases were well predicted by a dynamic mobility-weighted incidence index at a lead time of 1 to 6 months at the within-city level. The newly developed MI index captures the micro-level dynamics of human mobility and virus importation in a condensed form, making it useful for use in empirical regression models. The results suggest that human mobility is an important driver of the movements of incidence clusters within a city. We conclude that this novel early warning indicator has implications for dengue surveillance and early warning systems and can potentially enhance timely decision-making and coordination within the public health system.

## Supporting information

S1 Fig Total volume of tweets and users and population size in the 45 study neighborhoods in Yogyakarta municipality, Indonesia, August 2016—June 2018. (TIF)
