# Supplementary figures and images for: A combination of incidence data and mobility proxies from social media predicts the intra-urban spread of dengue in Yogyakarta, Indonesia
Source: PLoS Negl Trop Dis. 2019 Apr 15;13(4):e0007298. doi: 10.1371/journal.pntd.0007298 (PMC6483276; doi:10.1371/journal.pntd.0007298)

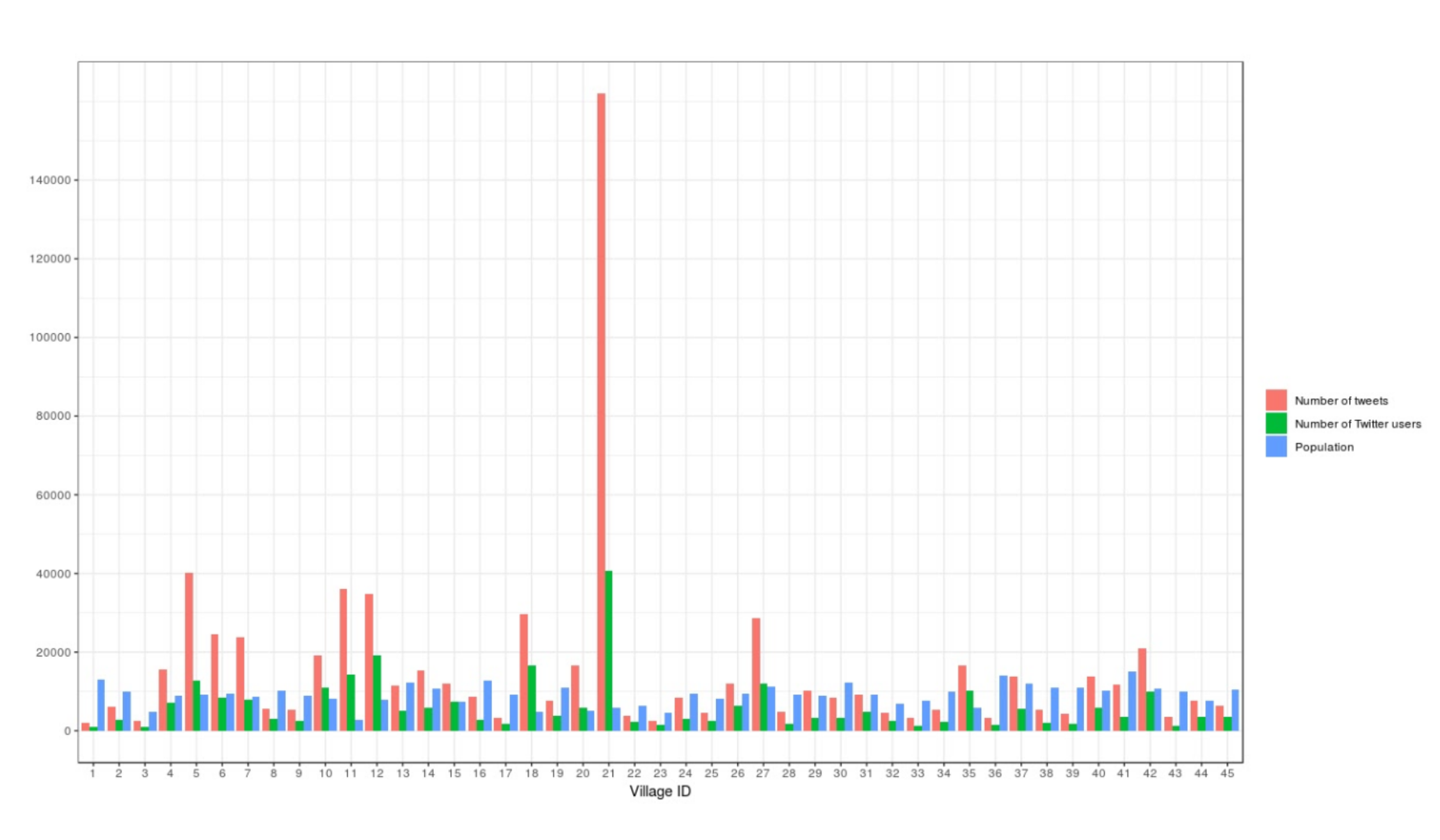

Supplement: S1 Fig — (TIF) [file pntd.0007298.s001.tif]
